# Navigating Hypersexuality in Bipolar: Insights from a Corpus-Assisted Discourse Analysis of Reddit Posts

**DOI:** 10.1177/00469580251338565

**Published:** 2025-05-21

**Authors:** Daisy Harvey, Paul Rayson, Fiona Lobban, Jasper Palmier-Claus, Clare Dolman, Steven Jones

**Affiliations:** 1 Lancaster University, UK; 2 Lancashire & South Cumbria NHS Foundation Trust, UK; 3 King’s College London, UK

**Keywords:** bipolar, hypersexuality, natural language processing, corpus linguistics, computational linguistics, discourse analysis, mental health

## Abstract

Bipolar is a severe mental health condition affecting at least 2% of the global population. Clinical observations indicate that individuals in elevated mood states, such as mania or hypomania, are more likely to engage in risk-taking behaviours, including hypersexuality. Characterised by an excessive preoccupation with sex, hypersexuality can lead to serious consequences such as sexual assault, unplanned pregnancies, and relationship breakdowns. Existing evidence suggests that opportunities for intervention in cases of hypersexuality in bipolar may currently be overlooked. This study provides insight into the lived experience of hypersexuality in bipolar using corpus-assisted discourse analysis (CADA) to qualitatively examine Reddit posts. The use of Reddit as a source of information in this study is particularly valuable because Reddit posts include narratives that may not be captured in traditional clinical settings. The candid, community-driven discussions found on Reddit can help identify gaps in care and raise awareness of the nuanced ways in which hypersexuality manifests and impacts individuals. Analysis of Reddit posts enables researchers to explore patterns, themes, and concerns that may be overlooked or underreported in clinical research, offering a more comprehensive understanding of the symptom of hypersexuality in bipolar. By employing keyword, concordance, and collocation methods, we identified 14 key thematic categories within the corpus and explored 4 of these in greater detail. The analysis showed that Redditors discussed hypersexuality within the context of bipolar symptoms, with many terms related to mood fluctuations and associated emotions. The diverse language used to describe sexual behaviours, along with references to shame and judgement, highlighted significant issues surrounding the discussion of hypersexuality in society and within healthcare settings. To enhance clinical practice, discussions about hypersexuality should be normalised and integrated into routine assessments, and healthcare professionals should receive training to address stigma.

## Introduction

Bipolar is a severe mental health condition characterised by recurring episodes of mania and depression which is thought to affect at least 2% of the global population.^1^ This paper refers to “bipolar disorder” as bipolar throughout in an effort to “drop the language of disorder.”^2^ The Lancet Commission^3^ describes that terms such as “disorder” which are used in the International Classification of Diseases (ICD-11)^4^ and the Diagnostic and Statistical Manual of Mental Disorders (DSM-5)^5^ can “victimise, criminalise, or misrepresent people with mental health conditions.” Previous research involving people with lived experience (PWLE) also suggests that people prefer to refer to their diagnosis as “bipolar.”^6^ This present research explores the presentation of hypersexuality as a symptom of bipolar using corpus assisted discourse analysis (CADA); ultimately suggesting recommendations to improve clinical practice.

Hypersexuality is difficult to define due to the absence of a universal standard for “excessive” sexual behaviour, which varies across cultures, individuals, and contexts.^7^ Research on hypersexuality in bipolar is limited, though broader studies explore it as an independent diagnosable condition.^7-11^ Bipolar UK^12^ defines hypersexuality as an excessive preoccupation with sex, often occurring during elevated mood states and leading to risky behaviour. It is a symptom that has been reported by people living with bipolar in previous (although limited) research,^6,13-17^ through lived experience reports^18-20^ and is included as an example of a risky behaviour during episodes of mania and hypomania in the DSM-5.^5^ We note that the term hypersexuality may be used by some individuals to articulate their personal experiences, even in the absence of negative consequences. While such self-descriptions may not align with the definition of hypersexuality adopted here, they represent meaningful aspects of those individuals’ lived experiences and deserve recognition.

Evidence from lived experience of bipolar highlights serious potential consequences which can result from hypersexuality, including “sexual assaults, unplanned children, vulnerability to sexually transmitted diseases, traumatic abortions and relationship and marriage breakups”^20^ These findings indicate that opportunities for intervention or support for people with bipolar experiencing hypersexuality may be being missed, and that healthcare providers may not have the awareness or training needed to provide the necessary support for this symptom. A recent survey on hypersexuality in bipolar conducted by the Bipolar Commission^12^ reveals that nearly 90% of participants reported experiencing hypersexuality, with over 50% of participants reporting 8 or more episodes of hypersexuality over the course of their lifetime.^18^ There is also research which describes stigma attached to hypersexuality^21,22^ and the discussion of sexual experiences with healthcare professionals,^19^ and a lack of qualitative research into the sexual behaviours of people living with bipolar.^13,18^

There have been calls for novel research “to address sexual symptomatology in bipolar within the context of current sexual, cultural and gender norms”^15^ as well as for research which provides better descriptions of hypersexuality to aid in early identification.^18^ A previous study which explored reproductive decision-making in bipolar exemplifies a dearth of research into sensitive topics related to sexual behaviours^23^ and demonstrates the use of Reddit for the collection of naturalistic data. Reddit affords users anonymity to discuss views which may not be “shared in clinic or in interviews”^23^ which leads to them being more likely to disclose “things they are unwilling to do or say in face-to-face settings.”^24^ This enables online users to talk candidly about sensitive and typically stigmatised topics such as hypersexuality. Reddit also affords the researcher a unique insight which extends beyond the scope of clinical interviews and self-report measures but raises ethical issues which are covered in detail below.

This study presents qualitative analysis of textual data from Reddit using corpus-assisted discourse analysis (CADA). Corpus linguistic methods have increasingly been applied to the study of data related to healthcare^25-28^ because they allow for the analysis of big data in a systematic way.^29^ Corpus linguistics uses real-world instances of language to explore patterns and meanings, providing a window into people’s emotional and cognitive worlds.^30^ In this study, we employ corpus methods to quantify linguistic patterns to answer the 2 primary research questions; (1) How is hypersexuality talked about by Redditors with bipolar? and (2) How could the findings of this research be used to improve clinical practice for people with bipolar?

By addressing these questions, the study aims to contribute to a deeper understanding of hypersexuality as a symptom of bipolar and how it is discussed within the online community. The study’s significance lies in its potential to inform both clinical practice and societal perceptions of hypersexuality in bipolar. By leveraging a corpus-assisted discourse analysis approach, this research also offers a novel methodological contribution to the field, demonstrating the utility of computational linguistics and text from social media to explore sensitive, often overlooked aspects of mental health. This conceptual contribution is timely, as increased awareness and understanding of hypersexuality could lead to better interventions and support for individuals with bipolar, particularly in clinical settings where such discussions are often taboo or misunderstood.

## Methodology

This study methodology implements CADA to analyse the presentation of hypersexuality on Reddit. This study adopted a pragmatist approach to research, whereby “pragmatic inquiry focuses on knowledge as the fallible and constantly revised product of experience”^31-33^ and where research design and methodology can take a multitude of forms. Maxwell^34^ uses the analogy of a “bricoleur” to describe pragmatist research, whereby the researcher employs using a combination of the relevant tools which are available to creatively solve a problem.

Due to the exploratory and interpretative nature of corpus analysis we first present our corpus methodology followed by an integrated section which describes the specific corpus methods and results. This is followed by an extended discussion of the research insights and clinical implications.

### Corpus Assisted Discourse Analysis (CADA)

The CADA methodology^35^ presented in this study builds on “traditional qualitative linguistic analysis by combining statistical overview analysis with close reading to bring added value.”^36^ Discourse analysis includes a wide variety of potential methodologies, and Flowerdew^36^ describes it as “one of the least clearly defined fields in applied linguistics.” In this study we take the view that discourse represents any text “above the sentence or above the clause.”^37^ Combining the approaches from corpus linguistics and discourse analysis enables us to mitigate the limitations of both methods – that discourse analysis may rely too heavily on the analyst’s preconceptions,^38^ and that corpus linguistics may rely too heavily on quantitative methods without in-depth explanatory power.^36^ In this study corpus linguistics provides us with the computational ability to analyse thousands of Reddit posts, which can then be “integrated with sustained qualitative interpretation” of these posts to “avoid reductive interpretations of words’ meanings.”^25^ Although research on mental health discourse is relatively under-represented, corpus-assisted methodologies have been described to explore recovery in bipolar,^39^ in the study of judgement patterns in bipolar^40^ and in the study of recovery in the anorexia community, depression, and diabulimia.^25^ Implementing a CADA methodology enables us to delve deeper into the “hidden” meaning in text in order to perform fine-grained qualitative analysis, which can yield significant insight into societal phenomena.^29^

### Corpus Creation

The Hypersexuality in Bipolar Reddit Corpus (HiB-RC)^41^ was created by scraping the social media site Reddit using the PushShift and Praw APIs (Application Programming Interfaces). Although Twitter was once called the “‘model organism’ for academic study” of social media, Reddit offers additional advantages. Posts from Reddit show how people respond to events as they unfold and offer a “qualitatively and quantitatively more expansive dataset,” meanwhile the structure of subreddits enables researchers to locate relevant data more quickly.^42^ There were 5 stages involved in corpus creation which are briefly described here. A detailed explanation of the corpus creation methodology can be found in Harvey et al.^41^

First, we scraped all of the posts from the 2 largest subreddits dedicated to bipolar: r/bipolar and r/BipolarReddit over 5 years between July 2017 and July 2022. The data collection took place in the UK but Reddit is an international site which is accessible globally, so posts were collected from English speaking Redditors regardless of location. Next, we used a pattern matching technique as has been implemented in prior research^41,43-45^ to detect Redditors who self-reported a diagnosis of bipolar (eg, “I have been diagnosed with bipolar”). To qualify for inclusion, diagnosis statements were required to (1) contain at least 1 condition term for bipolar, (2) match at least 1 inclusion pattern (ie, bipolar diagnosis of any type by a professional), and (3) not match any exclusion pattern (eg, self-diagnosis). We then scraped the entire posting history across Reddit for users who self-reported a diagnosis of bipolar. We note that there are limitations to using self-reported diagnoses as these have not been clinically verified within the dataset.

The posts were collected into a corpus which contained over 6 million posts from ~5000 Redditors – the Talking About Bipolar on Reddit Corpus (TABoRC). This resulted in a snapshot corpus that contains data that span 13 years, with the earliest post dating back to June 2009 and the latest submission date in August 2022. Fourth, we used a list of key words and phrases related to hypersexuality with a pattern matching methodology to identify posts within the TABoRC which referenced 1 or more of the hypersexuality terms – shown in Table 1. The hypersexuality search terms were created through an iterative process which involved training word embedding models on a corpus of interviews related to risk-taking behaviours in bipolar and identifying the most similar words in the Reddit corpus. The full methodology is provided by Harvey et al.^41^

**Table 1.** Hypersexuality Keyword Search Terms Derived from Word Embedding Model.

Hypersexuality keyword search terms
Hypersexual
Hyper sexual
Hypersexuallity
Hypersex
Hyper sexualised
Hyper sexuality
Oversexual
Hyposexual
Hyper sexualised
Hypersexualized
Overly sexual
Hyper sexualisation
Hypersexualization
Hyposexuality
Hypersexuality

Finally, we manually annotated these filtered posts for inclusion/exclusion to the hypersexuality corpus. This resulted in the HiB-RC: a corpus containing 2146 posts generated by 816 Redditors, which represents over 15% of the Redditors in the TABoRC. Compared to the results of the survey conducted by the Bipolar Commission which revealed that 90% of participants had experienced hypersexuality,^12^ this corpus is likely to be an under-representation of the number of Redditors who actually talk about hypersexuality due to the strict inclusion criteria that was used to identify posts about hypersexuality (discussed further in the Limitations).

In order to be included in the HiB-RC, posts needed to contain a statement which referred to a personal experience of hypersexuality (as opposed to posting about hypersexuality more generally). For example, a sentence such as “*I struggle with becoming hypersexual whenever I’m hypomanic”* would meet the criteria for inclusion, whereas a sentence such as “*Hypersexuality is a common symptom in bipolar”* would not.

### Validity

In this study, we aimed to enhance validity through a combination of qualitative rigour and quantitative reliability. When the HiB-RC was created, manual annotation of all posts was conducted to enhance the accuracy of data interpretation.^41^ A key validity measure was the second and third annotation of 10% of the corpus. This process helped to ensure inter-annotator reliability, minimising inconsistencies and reinforcing the credibility of the manual coding. By involving multiple annotators, the study reduced individual biases and confirmed the stability of interpretations.

Building on this foundation, the present study employed corpus linguistic methods, which are based on statistical analysis and are therefore reproducible. This quantitative approach provided a systematic way to analyse linguistic patterns, reducing the potential for subjective bias. Transparency in data selection, coding, and analysis contributed to the trustworthiness of the findings.

### Reflexivity

As the lead researcher conducting this study, I acknowledge the role of my background, perspectives, and methodological choices in shaping this study. My academic expertise in corpus linguistic analysis and qualitative research has informed my approach to examining how hypersexuality is represented and constructed in language. However, I recognise that my own assumptions about mental health, sexuality, and societal discourses may have influenced the framing of this research.

Given the sensitivity of the topic, I remained mindful of potential biases and took deliberate steps to ensure analytical rigour. The corpus-assisted methodology provides an empirical basis for identifying linguistic patterns, helping to mitigate interpretative subjectivity. However, I acknowledge that the selection of texts, keyword choices, and thematic categorisations involved subjective decisions that could reflect my own positionality.

Furthermore, I was conscious of the ethical implications of analysing discourse related to a stigmatised aspect of bipolar. I approached the data with an awareness of how language can reinforce or challenge dominant narratives, striving to provide a nuanced and responsible interpretation. By maintaining a reflexive stance throughout the research process, I aimed to produce an analysis that is both critical and ethically sensitive.

### Ethics

Our research methodology has been strongly influenced by ethical considerations, and we have sought guidance from multiple sources including the British Psychological Society (BPS), the British Sociological Association (BSA), the UK government^46-48^ and academic research guidelines.^43,49-52^ This study was conducted as part of a PhD thesis on the topic of risk-taking behaviours in bipolar, and the framework methodology for the PhD was reviewed by a panel of lived-experience advisors through Lancaster University Spectrum Connect. Ethics approval was granted for the project by Lancaster University in December 2021 (FHMREC21042).

Reddit has over 50 million daily users and 100 000 *active* Subreddits in 2024^53,54^ and research has shown that the anonymity afforded by social media sites enables users to self-disclose on sensitive topics that they may otherwise find difficult to talk about.^24^ From a legal perspective, while Reddit is inherently an anonymous platform, it is possible that users employ the same username on other social media sites or platforms. Consequently, it is good practice to regard the information collected from the site as personal data. As researchers we acknowledge that the Reddit posts used in our study contain sensitive information, and we have only collected data that is available in the public domain. We did not seek informed consent from the Redditors whose posts were included in our corpus because this would have been impractical considering the number of posts and Redditors in our initial corpus from where the hypersexuality posts were filtered (~5000 Redditors in the *Talking About Bipolar on Reddit* Corpus: TABoRC). We note that Reddit users are made aware that their posts are publicly accessible through Reddit’s Terms and Conditions.

Employing corpus methods to analyse Reddit posts allows us to empirically study language and gain insights into a potentially hard-to-reach population. We recognise the serious ethical implications of collecting such sensitive information but believe that the improved understanding and awareness derived from analysing Reddit posts is significantly valuable for individuals who experience hypersexuality as a symptom of their bipolar diagnosis. We have adopted procedures reported in guidelines^46,49,50^ to protect the anonymity of the Redditors in our dataset. We have masked the usernames in this dataset (ie, created alternative alphanumeric usernames for each Redditor in the dataset) and have only included paraphrased and depersonalised quotes in research outputs. Where we have presented paraphrased quotes, we verified that Redditors could not be re-identified based on an internet search of the reworded quotes. Using these methods, we have strived to maintain the privacy of the Redditors included within our corpus as much as possible. While we acknowledge the limitations of using online-sourced data, we also recognise the unique insights that such data analysis can offer.^18,19^

Using Reddit as a primary data-source is not “wholly problematic or must be ceased,” but “careful handling and anonymisation of such materials is of paramount importance for maximising ethical research practice going forward.”^52^ The analysis of this corpus was conducted using Sketch Engine^55^ which has ISO27001 certification in cyber security, and where data is only accessible on an individual user basis in a password protected environment. In terms of data dissemination, a redacted version of the HiB-RC is available from the UK data service as requested by the funder of this research (the ESRC). The redacted corpus includes only the post IDs generated by Reddit for the posts which form the corpora, which can be used to retrieve the posts using an API. The corpus will only be disseminated upon request on a case-by-case basis to researchers with an institutional email address. This complies with Article 17 of the UK GDPR and an individual’s rights to data erasure because any content which has been removed since the creation of our datasets will appear as [removed] upon retrieving the post ID using an API. Our full data availability statement is available at the end of this paper.

### Corpus Methods and Results

#### Observing Key Thematic Categories in the Corpus

As is typical of CADA^56^ and using the keyword to categorisation methodology provided in previous research as a framework,^57-59^ our analysis began with the statistical identification of keywords and key phrases in the corpus using Sketch Engine.^55^ Keywords were identified by comparing the focus corpus to a reference corpus to calculate words which occurred statistically more often in the focus corpus. Within this study the focus corpus is the HiB-RC and the enTenTen21^60^ serves as a reference corpus, which is an English corpus made up of texts collected from the Internet and contains around 50 billion words. In Sketch Engine keyness is calculated using the simple maths method, which compares the relative frequencies of the words in both the focus and reference corpus to produce a keyness score. This score essentially translates as “*the keyword is X times more frequent in* corpus *A than* corpus *B*.”^61^ We limited our keyword list to the first 1000 keywords and stipulated that words and phrases had to appear in at least 5 texts within the corpus to be considered key, that is, be used by at least 5 Redditors to ensure that usage was not idiosyncratic to 1 user. We also stipulated that a word or phrase needed to have a keyness score of at least 3, that is, be 3 times more likely to appear in the focus corpus than the reference corpus. After generating the key terms, we manually identified meaningful clusters and grouped similar lexical items to provide an insight into the key thematic categories frequently discussed by Redditors in the HiB-RC. These categories and salient keywords are provided in Table 2. In total 400 key terms were grouped into 14 categories, from a total of 9836 unique lemmas in the corpus, prioritising content words (eg, nouns, verbs adjectives) over grammatical words.

**Table 2.** Key term Categorisation of Thematic Categories in the HiB-RC.

Number	Key thematic category	Examples of keywords
1	Diagnosis and symptomatology of bipolar	bp2, psychosis, bp1, psychotic, hallucinate, symptom, dissociate, schizoaffective, bp, bpii, illness, dysphoria, hormonal, cycling, mentally, trigger, fluctuate, mental, hormone, puberty, relapse, meltdown, grandeur, flashback, escalate, onset, breakdown, sick, emotional, baseline, appetite, talkative, codependency, insomnia, manic, hypomanic, hypomania, mania, hyper, hypo, depression, mood, upswing, elevated
2	Sexual behaviours	Urge, horniness, arousal, horny, libido, hypersexual, hypersexuality, masturbate, masturbation, cheat, fwb, sex, tinder, hookup, porn, promiscuous, cheating, binge, fantasise, fuck, sexting/ texting, kink, cheated, orgasm, fetish, flirty, bdsm, boner, kinky, cheater, infidelity, unfaithful, promiscuity, threesome, stranger, cum, prostitute, crush, condom, orgy, seduce, hoe, lust, fling, penis, sexy, erection, climax, stimulation
3	Pharmacological	Lamictal, medicate, med, seroquel, latuda, ssri, unmedicated, abilify, wellbutrin, lamotrigine, antipsychotic, lexapro, zoloft, vraylar, antidepressant, stabiliser, zyprexa, adderall, lithium, geodon, vyvanse, depakote, ssris, prozac, klonopin, medication, olanzapine, xanax, benzo, anti-depressant, welbutrin, effexor, testosterone, dosage, pill, dose, prescribe
4	Sexuality/sexual orientation	Sexuality, asexual, polyamory, polyamorous, monogamous, monogamy, sexually, sexual, demisexual, bisexual, poly, demi, bi, sexless, lesbian
5	Emotions and feelings	Irritable, impulsivity, euphoric, delusional, paranoid, insatiable, restless, obsessed, reckless, numb, hopeless, extroverted, agitation, destructive, compulsion, craving, uncontrollable, anxious, terrifying, crippling, overthinking, debilitating, intense, erratic, nonstop, hyperactivity, rage, fixate, all-consuming, uncomfortable, horrible, hurtful, rollercoaster, energetic, panic, scared, giddy, hurt, unhealthy, angry, itch, unstable, inflated, frustrated, overdrive, unbearable, overwhelmed, energise, atypical, outgoing, exhausting, hate, insecure, irrational, miserable, stressful, pointless, afraid, emptiness, temper, lonely, struggle, chaotic, vibrate, devastate, dangerous, upset, sad, alone, worthless, hard, confused, unhappy, worry
6	Therapy and treatment	pdoc, psychiatrist, psych, therapist, dbt, appt, inpatient, diagnose, misdiagnose, undiagnosed, diagnosis, untreated, psychologist, therapy, EMT, psychiatric, doc, doctor, appointment, er, hospitalise, hospitalisation, induce
7	Relationships	Boyfriend, coworker, bf, gf, ex, ex-husband, girlfriend, relationship, partner, husband, roommate, fiance, spouse, mom, romantic, married, guy, divorce, marriage, friend, faithful, romantically, intimacy, breakup
8	Shame and judgement	Ashamed, shitty, slutty, slut, disgusted, repulse, asshole, regret, crazy, weird, loathing, sketchy, embarrass, guilt, awful, shame, freak, insane, disgust, embarrassed, cringe, taboo, disgusting, embarrassment, forgive, stupid, repress, terrible, bad, inappropriate, stigma, shameful, humiliate, mess, dumb, sorry, cry, judgement, horrify, embarrassing, irresponsible, ruin
9	Sleep	Sleep, sleeping, tired, asleep, wake, awake
10	Risk-taking/impulsivity	Hyperfocus, overspend, self-harm, meth, drunk, stimulant, risk-taking, drinking, spending, inhibition, cocaine, weed, suicide, coke, alcohol, alcoholism, piercing, broke, hell, drink, drug, heroin, tattoo, sabotage, addict, suicidal, STD, STI
11	The self	Myself, introvert, feel, self, brain, realise, self-esteem, superpower, invincible
12	Management strategies	Daylio, sober, iud, stable, vibrator, dopamine, quit, cope, hobby, distract, supportive, abstain, happily, calm, stability, gym, stabilise, toy, motivation, rehab, mindfulness, helpful, outlet, amends
13	Trauma and abuse	Rape, abuse, csa, traumatise, trauma, groom, molest, abuser, narcissist, abusive, traumatic, manipulative
14	Comorbidity	bpd, adhd, schizophrenia, ptsd, ocd, cptsd, anxiety, mdd

Our methodology shares similarities with traditional thematic analysis^62^ but differs in key aspects. Like thematic analysis, our corpus approach provides a detailed, data-informed account with an inductive, semantic, and experiential focus.^63,64^ However, while thematic analysis follows a structured coding process, we diverged from this by using keyword analysis to guide the inductive process of creating thematic categories. After obtaining the keywords, we followed standard corpus linguistics practice, grouping them thematically based on their semantic properties and contextual usage. We refer to these groupings as thematic categories, distinguishing our approach from “pure” thematic analysis as outlined by Braun and Clarke.^63^ This methodology aligns with established research practices and builds on similar approaches used in previous studies.^57-59^ By leveraging corpus-linguistic methods to highlight salient data statistically, our approach enables a more efficient and replicable qualitative analysis of a larger dataset.^56^ Table 2 presents key thematic categories based on Redditors’ lexical choices.

While all 14 identified categories offer valuable insights, an in-depth analysis of each is beyond the scope of this paper. Therefore, our decision to focus on 4 key thematic categories was guided by an inductive qualitative approach, allowing themes to emerge organically from the corpus rather than being predetermined by existing theories.^62,65^ Our selection was shaped by the richness of discourse, clinical significance, and potential to address critical gaps in the literature. This process was further informed by reflexivity and co-production, as 1 of the co-authors, who has lived experience of bipolar, contributed to the development of this research. Reflexivity in qualitative research highlights the importance of integrating diverse perspectives, and co-production ensures findings are grounded in real-world experiences.^66^ By combining corpus linguistic methods with an experiential perspective, our analysis balances empirical rigour with the nuanced realities of hypersexuality, enhancing both the validity and clinical relevance of our findings.

The 4 selected themes are outlined below:

1. Sexual Behaviours: Understanding specific sexual behaviours associated with hypersexuality is essential for situating this symptom within the broader framework of bipolar. This theme also provides insight into how individuals perceive and experience these behaviours, which can inform more individualised care strategies.
2. Shame and Judgement: The intersection of hypersexuality with societal stigma often leads to profound feelings of shame and judgment^21,67-69^ which are underexplored in the literature related to hypersexuality in bipolar. Naturalistic data from Reddit provides unique insights into this experience.
3. Trauma and Abuse: Hypersexual behaviour during manic episodes can lead to traumatic experiences, either directly through the behaviour itself or through the social repercussions that follow. Research also suggests a potential link between childhood sexual trauma and later hypersexuality,^22,70^ making this an especially complex and underexamined theme.
4. Therapy and Treatment: Understanding how individuals with bipolar experience therapy provides insights into barriers and facilitators of effective treatment. This theme contributes to discussions on developing more targeted, compassionate, and stigma-sensitive mental health interventions.

By prioritising these thematic categories, we ensure our analysis contributes meaningfully to existing knowledge. Additionally, our approach captures the complex behavioural and emotional dimensions of hypersexuality, which are often overlooked in clinical and academic discussions.

#### Concordance and Collocations

Concordance lines enable us to situate a key word or term within its original context, and collocations measure the association between words based on patterns of co-occurrence that is, determining whether specific words have a strong preference to occur together.^56^ Thus, these methods can help to reveal the linguistic patterns which contribute to the construction of hypersexuality on Reddit. Collocations were identified using the WordSketch tool in Sketch Engine, which provides a summary of a word’s grammatical and collocational behaviour. WordSketch organises collocations into categories (eg, modifiers, objects, adjectives) based on a LogDice score which represents the strength of a collocation; where a higher score represents a higher strength collocation.^71^ The findings of this analysis are described narratively below.

### Sexual Behaviours

Collocational analysis reveals that the adjective hypersexual is modified by words such as *extremely, incredibly, ridiculously, intensely, painfully* and *insanely*, demonstrating the intensity of hypersexual experiences. Meanwhile, the “*hypersexual* and/or” results of the WordSketch tool demonstrate that hypersexual occurs together with or in similar contexts as items such as *manic, hypomanic, impulsive, talkative, euphoric, irritable*, and *insatiable*, suggesting that hypersexuality collocates with an elevated mood and common symptoms of mania and hypomania including rapid speech, feelings of elation and impulsivity.^5^ The noun sex is modified by words such as *casual, risky, unprotected, unsafe* and *rough*, suggesting that some Redditors experiencing hypersexuality may engage with unsafe sexual practices during hypersexuality. Paraphrased concordance examples for the keyword sex are shown in Table 3.

**Table 3.** Paraphrased Concordance Examples for the Keyword Sex.

ID	Left context	KWIC (keyword in context)	Right context
04f6a516-ac58-42c9-bc97-5bfa11397d10	I end up participating in risky casual	sex	, which is something I wouldn’t do normally.
11cbb27b-a25e-4825-875d-5f8538b0b6e0	I have engaged in casual	sex	with numerous strangers in high-risk situations.
a11ee3e5-5617-4316-9330-052838ab2f53	A LOT of risky, absurd	sex	/hooking up for over 6 h a day, usually even more.
c02acab8-28a0-4e8e-87b7-4867657a3d50	My hypersexuality showed up as obsessive, insatiable masturbation and constant risky	sex	. I literally could not stop myself from doing it.
3f7f8265-b052-4634-8e3c-67738a39a8e2	I got involved with drug users and thieves and had unprotected	sex	in an abandoned meth house.
b5ed3174-7991-45b7-93f4-5b29149dadd9	I keep drinking and having unprotected	sex	(hypersexual) every night.
b8187f44-a78a-4a2f-92cb-7003102af933	I made the worst mistake by receiving unprotected oral	sex	from a prostitute during a manic episode.

Collocational analysis also reveals that some Redditors describe being *insatiably, distractingly, unbearably, uncomfortably* and *uncontrollably* horny, with some Redditors using modifiers such as *continuously, compulsively, excessively*, and *constantly* in relation to the verb masturbate. A number of keywords related to sexual behaviours were described by Redditors including *hookup, cheating, prostitute, orgy, hoe, threesome, tinder, porn* and *fwb* [friends with benefits]. Concordance examples for some of these keywords are provided in Table 4 to demonstrate some Redditor experiences.

**Table 4.** Paraphrased Concordance Examples for Sexual Behaviour Keywords.

ID	Left context	KWIC (keyword in context)	Right context
00384b4e-f73f-4025-9b31-12641909be60	I began behaving oddly and having intense	hookups	and wide and unusual interests.
24eef637-0984-4cb6-b53f-a5a76c9cbfea	I ended up just browsing	hookup	sites all day and night trying to get laid.
41f975b9-6d7a-4757-bdcc-05e360acda77	Every time I go through with a	hookup	I can’t help but feel utterly disgusted and disappointed with myself.
4325a03f-6973-4901-912b-4839fec46606	I start doing risky things like picking up	prostitutes	. . .I know it’s bad if you are truly in love.
83416d1c-b50a-4df1-9cfd-bddad4ca847e	For me, this involved strip clubs,	prostitutes	, and orgies.
53a78634-1ba3-4258-8381-41ef48f84abe	I agreed to a	threesome	. To this day, I’m ashamed of that.
a1c6046b-5a63-42af-87d3-5c24eec84e0d	My past is filled with one-night stands,	threesomes	, etc. I definitely used sex as a form of self-harm.
33fb4f50-c9d8-407b-aebf-685f3a4d1be5	I knew	Tinder	can put women in those situations and yet I used it anyway.
e4498466-c485-460b-be0f-e9afbfa94c3e	I get hypersexual when I have hypomania, so deleting	Tinder	helps prevent impulsive hookups.
146d4de3-9ab3-4fe2-abbc-f136366de60c	I was hypersexual and watching hours of	porn	, I cheated and began webcam modelling.
2a02b328-910e-4f8b-8db3-48f0034930aa	I’m constantly looking at	porn	and I don’t think I’d say no to anyone.

In relation to the verb cheat, collocation analysis reveals diverse experiences were reported by Redditors, with *never* appearing as the strongest collocational modifier, but other lexical items such as *invariably, continuously*, and *repeatedly* also being used. A number of Redditors discuss that having bipolar and/or hypersexuality is not an excuse for cheating, whilst other Redditors express that they struggle with fidelity during periods of hypersexuality. Concordance examples are provided in Table 5.

**Table 5.** Paraphrased Concordance Examples for the Key Lemma Cheat.

ID	Left context	KWIC (keyword in context)	Right context
1120fcd3-1886-47fa-8201-f91e2fb658b2	I need a lot of sex whether I’m on or off meds but I’ve never	cheated	on someone.
9bf2fcb5-c26e-4bf8-9f99-cd61240e85f4	I have hyper sexuality but I have never	cheated	on anyone in any way.
e671a3af-8aa5-409b-91b0-934f8191c335	Hypersexuality is a symptom a lot of us will experience but cheating is a choice. I’ve never	cheated	on my husband when hypersexual
bc78df3c-2021-42e7-b964-49df3a685982	I don’t have sex with anyone else, but I invariably	cheated	on previous partners.
77480a5f-8795-4a60-82e7-3d6a6c5df277	All he said to me when I said I was continuously	cheating	on my ex-husband was to divorce him
175d7e5d-d087-4355-becf-f847a6ed420c	I’ve	cheated	in pretty much all of my relationships.
23f856e4-4099-4f30-a002-bc70d27b5d9c	Even when I’m hypersexual I don’t	cheat	because i don’t feel this is acceptable.
347c2bd9-e743-4424-a54e-ee3977009f33	I personally did	cheat	a lot on my partner during mania.

There are a number of posts which describe strategies that Redditors use to manage hypersexuality, including using sex toys and masturbating to prevent riskier sexual experiences. Some posts also reference that taking or changing medications has helped Redditors to manage their hypersexuality. Concordance lines demonstrating examples of such posts are presented in Table 6.

**Table 6.** Paraphrased Concordance Examples for Sexual Management Strategies.

ID	Left context	KWIC (keyword in context)	Right context
171cc1fb-46a1-449e-9b8e-959e019423d5	I could	masturbate	for hours but still feel frustrated
2a02b328-910e-4f8b-8db3-48f0034930aa	I	masturbate	until it hurts.
5cf6f77d-1db7-46b6-9c5b-bddb80b5ae06	During COVID I	masturbated	5 times a day or more.
d8ac9037-1dda-4de4-8043-536442f8aead	These days my mania is hypersexuality. Meds have made it tolerable and I just use a	vibrator	or go to my partner
d8ac9037-1dda-4de4-8043-536442f8aead	Before I was on this dosage my hypersexuality would involve meeting strangers and having one night stands. These days I’m able to handle it using a	vibrator	.
6ee74d17-6973-4d28-a01b-bd7fd306914e	I deal with my hypersexuality by using	toys	, it means I can do it as many times as I like without the risk of STIs.
c5ffaa5e-754d-4b87-acf5-b75ae8db8b9a	I would advise trying to find pleasurable ways of coping; porn,	toys	, or a trusted partner.

We also created a sub-corpus of posts which included the terms hypersexual and hypersexuality and searched within these posts for the phrase “didn’t know”. We identified that a number of Redditors describe that they engaged in risky sexual behaviours prior to understanding that they were experiencing hypersexuality or being aware that hypersexuality is a symptom of bipolar. Examples of these concordance lines are provided in Table 7.

**Table 7.** Paraphrased Concordance Examples for the Phrase “Didn’t Know.”

ID	Left context	KWIC (keyword in context)	Right context
35c19ccf-d57f-475f-915a-0d4589fa310e	I didn’t know I was bipolar then, I	didn’t know	I was manic, and didn’t know the word hypersexual.
44cfd82e-311c-4988-b910-4193c22a59e9	I	didn’t know	what mania was, so when I became really delusional and hypersexual I didn’t know anything was wrong with me.
4ebe7ad8-bbce-4a9d-aecc-6901f0aa6d4f	I really	didn’t know	I was hypersexual at the time until I looked at the disorder in more detail.
5d17cff1-9df4-45df-a641-7a72e5561260	I did very risky things that I’m not proud of because of hypersexuality. I	didn’t know	it was a symptom until much later in life but now it all makes sense.
71f52d1c-e096-4c55-a758-66fee880f7df	I	didn’t know	until this year but I have hypersexuality. I’m just looking for a quick fix and I’m not picky with my partners.
d7588088-7522-45d2-acad-bfd72ce57b00	Hypersexuality has been eye opening. I	didn’t know	it was linked to symptoms of hypomania, it really has explained a lot.

### Shame and Judgement

Redditors in the corpus express shame and judgement using lexical items such as ashamed, disgust, regret, embarrassing, guilt, stigma, taboo, and shame. Collocations for ashamed, shame, and guilt are demonstrated in Table 8. The modifiers presented in Table 8 demonstrate that Redditors express strong and frequent feelings of shame, resulting in compounded feelings of regret, worthlessness and humiliation. We note that the noun shame is used in relation to STIs as well as mania-induced behaviour.

**Table 8.** WordSketch Collocates for Keywords Related to Shame and Judgement.

Key thematic category	Key term	Modifiers of “X”	“X” and/or
		Collocate	Collocate
Shame and judgement	Ashamed (adjective)	So	Embarrassed
		Extremely	Guilty
		Very	Disgusted
		Not	Uncomfortable
			Sad
	Guilt (noun)	Subsequent	Shame
		Shame	Remorse
		Insane	Worthlessness
			Stimulation
	Shame (noun)	Mania-induced	Guilt
		Unbelievable	Embarrassment
		Utter	Regret
		Deep	Humiliation
		Intense	Disgust
		Extreme	Cringe
		Little	STIs

In Table 9 the use of the word stigma suggests that Redditors have experienced stigma related to mental illness generally, as well as stigma associated with sexual behaviours and stigma experienced within relationships from partners. The term taboo is used by Redditors to express frustration at how sex and hypersexuality is viewed by society and healthcare professionals, with 1 Redditor also describing that hypersexuality is a taboo even within the bipolar community. Redditors describe feelings of shame related to hypersexual behaviours which can pervade months or years after a period of hypersexuality.

**Table 9.** Paraphrased Concordance Examples for Keywords in the Key Category of Shame and Judgement.

ID	Left context	KWIC (keyword in context)	Right context
51ac7e73-76ef-47ae-a271-db01bd632c74	It leaves you to come to terms with what it means to be bipolar. Not to mention the	stigma	of things like hypersexuality, the sense of shame you might be made to feel for that.
7e24e264-a133-43ed-a5e2-c33a14dd9160	I don’t tell anyone that I have bipolar because of the	stigma	. I sometimes tell people I have depression because I think that is less stigmatised.
eb79dc05-0972-4039-8648-9b5e51ffff10	Hypersexuality is something I literally don’t talk about with my own husband. Because he himself has	stigma	around it. So. . .would be pretty cool for that to not be a thing. And the only way is to start normalising it.
93ff9b5e-8694-4ccd-8a04-637c86ba25f5	I’m ashamed of acting like that and I wouldn’t tell my doctor because sex is	taboo	.
b3a83d10-554e-43b9-aea7-e12a770132df	I want to cheat because it’s	taboo	and something you shouldn’t do.
bf6a2f14-eac7-4e2f-8185-41925b85729a	My biggest problem is hypersexuality but this seems to be	taboo	to talk about even within the bipolar community.
d52010c8-60af-45b9-b047-6d7a09ca232d	I wish therapists talked more about hypersexuality, it’s like a	taboo	subject that I had to learn about on my own.
e0e97bdd-8798-44c7-9611-f648abc5e38b	Is hypersexuality part of being bipolar - why isn’t it talked about and so	taboo	, because it’s so painful, how do I bring that up to my psychiatrist?
38b47f6e-a21e-472b-a4ec-31016c139b27	I struggle with the memories of some of the things I did especially when I was hypersexual, it just causes me so much	shame	even a decade later.
4cd749d0-a42d-47ff-8ffa-7c5115dc82e8	Hooking up with strangers when I was experiencing hypersexuality evokes the most	shame	and embarrassment.
f56891f8-d878-4e1f-9f72-062bd3080294	I had sex with four people in one night. I’m so	ashamed	. I was hypomanic/ manic so I can’t remember everything I did.
3a4e9207-7553-499b-9b5e-cc20517255d3	Cheated while manic and very hypersexual and I’m still dealing with the	guilt	and anxiety months later.
facb09b3-6549-460c-b482-bb4b1a21f932	I’ve been hypersexual for 3 months and it’s awful. It’s the most	guilt	creating thing. I tend to hook up with random guys.

### Trauma and Abuse

The concordance lines for posts which include keywords related to trauma and abuse are presented in Table 10. Exploration of the keywords presented in Table 2 reveal that a number of Redditors describe childhood experiences of abuse. Analysis of keywords such as abuse and trauma show that some Redditors directly link this to hypersexuality in later life, as well as describing trauma as a result of events experienced during hypersexuality. A number of Redditors specifically describe being molested or groomed as a child and some discuss whether hypersexuality is a “tool” they use to cope with the sexual trauma they experienced when they were younger.

**Table 10.** Paraphrased Concordance Examples for Keywords in the Key Category of Trauma and Abuse.

ID	Left context	KWIC	Right context
11cbb27b-a25e-4825-875d-5f8538b0b6e0	I frequently experience episodes of hypersexuality which are made worse by the	abuse	I received as a child. Reddit is the only place that knows about my sexual exploits.
11bead71-c117-453c-8812-4e49267c0b95	It’s trauma related to sexual	abuse	in my past. I’m manic at the moment and I’m completely obsessed with my sexual trauma.
14e5bc97-be05-4fc1-aef0-74ee6931a941	I’ve been promiscuous and hypersexual my whole life, I’ve had problems with bed wetting. My therapist said these could all be symptoms of sexual	abuse	.
6b48b077-94d5-41bc-8f94-3b41e4b06ece	I masturbated constantly and I didn’t remember my	abuse	at this time, I was religious and thought I was being punished by God.
7f536231-e84b-4e30-9803-bedc26aecb82	I have repressed memories of being raped as a child and my dad sexually	abusing	me as a teenager.
44e1a35b-4396-4215-8e30-90ac18c37359	I was	molested	between the ages of 4 to 7. It’s awful to remember.
03713692-4072-4e65-829f-c276b6c8a6f1	I believed I was	raped	because I was promiscuous. Before Reddit I was very alone with all of these feelings.
0fed29bd-efa9-4123-ac7c-35c2ad5c8054	In another manic episode with hypersexuality there was a lot of risky behaviour and I was	raped	during this time.
3a94f59a-d400-4de2-a201-8d4e5a66266d	I was shocked by my bipolar diagnosis but I know it’s true because of psychotic episodes and hypersexuality. I was	raped	earlier in the year, I’m so scared of being hurt again.
79061f1b-f317-46f9-8cf1-b7334d6c8341	I have bipolar and I’m manic. I hadn’t become hypersexual yet but then I was	raped	after a week.
9a0c79bc-ff81-4592-a585-1ac1a9285eb4	I had multiple sexual partners in a day during mania and also	rapes	. It felt like sex was controlling me.
b608a64f-a2ad-4ea4-a296-b68303af6690	I kept getting into dangerous sexual situations. I visited a sex club and was	raped	. Afterwards I always feel like shit.
56cbe24f-5111-4b30-a65e-6f94657b255a	I was diagnosed with	PTSD	due to something traumatic that happened during a mixed episode. I made reckless decisions and ended up being assaulted.
1bdb9640-f348-4253-9952-ecead58cb8f8	I’m starting to obsess about sex, I think I’m going to do something that I’ll regret which will be	traumatising	afterwards.
f04ca4e1-63a7-4c89-b830-e2861d82e393	I became hypersexual as a result of	trauma	, sometimes the brain needs to heal by reliving something.

Concordance analysis of the keyword rape reveals that some of the posts relate to events that occurred during childhood and pre-diagnosis, while others describe being raped whilst being in compromising sexual situations. A number of the posts describe that they have rarely shared these experiences beyond Reddit.

### Therapy and Treatment

Concordance analysis of posts containing keywords related to treatment and therapy reveals a wide range of healthcare experiences, as summarised in Table 11. Many Redditors express dissatisfaction with their interactions with healthcare professionals, highlighting issues such as psychiatrists who rush through appointments, difficulties in finding a supportive or effective psychiatrist or therapist, and instances of unethical behaviour, including inappropriate comments about hypersexuality. Numerous posts also describe healthcare professionals as being uncomfortable discussing hypersexuality, with 1 Redditor noting that the topic is only addressed if they bring it up themselves, while others report receiving minimal advice or having the issue dismissed. Additionally, several posts describe personal feelings of shame and embarrassment, which lead to individuals withholding information about their sexual behaviours from their healthcare team. This lack of disclosure often results in confusion and distress, leaving Redditors unsure of how to manage the symptom and struggling with negative emotions.

**Table 11.** Paraphrased Concordance Examples for Keywords in the Thematic Category of Therapy and Treatment.

ID	Left context	KWIC	Right context
16807da2-a099-4c75-a1c9-622f352cd017	I thought my	psychiatrist	was good the first time I saw him. But last week he only spent 5 min with me and clearly wasn’t listening to what I was saying.
d666c45f-8206-4a00-baee-3c2d1c05af12	I think my	therapist	might have a crush on me. He seems almost too interested when I talk about hypersexuality. It feels like he’s checking me out.
7636dccc-252f-42a6-b94b-5ee1c048ff7a	I’m fairly recently diagnosed and I’m really struggling to find a	therapist	that doesn’t suck. The last one didn’t diagnose me with anything but gave me loads of medication which basically ruined my life.
423e808f-a53f-4d2f-91b2-421ecc781e50	I’m hypomanic on the psych ward and my hypersexuality is getting worse. I’ve told my	psychiatrist	multiple times but there’s never a response.
d52010c8-60af-45b9-b047-6d7a09ca232d	I wish	psychiatrists	and therapists talked about and dealt with hypersexuality. It’s like it’s taboo, I had to read about it myself it was never brought up in appointments.
feea1285-8eaa-4f9a-a9f9-4cc626dbf9a6	I’ve had a number of affairs since I was diagnosed and I feel terrible. I tend to hide this information from my	therapists	because it’s so shameful. I really don’t know what to do anymore.
d52010c8-60af-45b9-b047-6d7a09ca232d	I have hypersexuality, but the weird thing is that	therapists	are so reticent to talk about it. I’ve never had one who brought it up, not even in hospital. When I bring it up they get uncomfortable and brush over it.
ddde965c-a5de-4078-b763-10113a32389e	I feel so isolated by my hypersexual feelings. No one understands, not my partners, friends or my	therapist	. It means so much to know that it’s not just me.
58e07c17-2340-47de-aff8-6cde08a56d5a	My new	psychiatrist	explained to me what hypersexuality is. I wish that I’d got therapy earlier.
9298e306-c9dc-4ff5-a276-b25dc5769c97	Now I’m aware it’s a symptom so when I feel a bout of hypersexuality I talk to my	psychiatrist	to increase my medication. I’m not cured but at least I’m in control.
9298e306-c9dc-4ff5-a276-b25dc5769c97	One of the first questions my	therapist	asks me is whether I’m staying off dating apps – it’s her way of gauging how I’m doing.
eb1e025b-7ead-48ee-80af-0921afa5497e	I know that I become hypersexual during hypomania. I’m on proper medication and I’ve learned coping skills through	therapy	so I can now deal with these urges in a much healthier way.
9a0c79bc-ff81-4592-a585-1ac1a9285eb4	I was in	therapy	for years before I was comfortable talking about some of the worst things I’ve done when I’m hypersexual. I felt so ashamed but it made me feel much better to get it out and see their neutral reactions.
0d2165a3-0608-4c76-b2a4-8f5f2ef24c36		DBT	Therapy has saved my life. It’s about emotional regulation and how to deal with life when things get hard – coping tools for my emotional toolbox.
b153dd08-9279-4f3c-ab5f-aa33a43f9636	I think noticing behaviours early and doing	DBT	exercises could help. If we start people thinking about their mental health early we could save people from pain – breathing exercises, small goal setting, planning for episodes and how to take care of yourself.
a5c1bb00-3bc3-4608-bd65-53a54ccbb25e	I was diagnosed with bipolar II this week. I was in denial because I didn’t think my symptoms were severe enough as I was never	hospitalised	.
a8df2128-8df5-4f61-b0a1-beb7c277a82a	I was bipolar II as a teenager but not diagnosed until I was	hospitalised	In my 30s. I was very hypersexual and doing inappropriate things.

There are also posts which express positive sentiment in relation to healthcare experiences, with Redditors describing that therapeutic intervention has enabled them to become more aware of symptoms and develop coping strategies to remain in control during hypersexuality. Redditors also frequently describe that a combination of medication and therapy has been beneficial, and some Redditors describe that specific interventions such as Dialectal Behaviour Therapy (DBT) have enabled them to be more aware of symptoms like hypersexuality and develop emotional tools to be able to manage patterns of behaviours.

A number of Redditors describe their experiences with diagnosis in relation to hypersexuality. One Redditor states that they were hypersexual as a teenager although they were not diagnosed with bipolar until their 30s after a period of hospitalisation, whilst a number of Redditors describe that they weren’t aware that their symptoms could be indicative of bipolar because they hadn’t been hospitalised – eventually leading to a diagnosis of Bipolar II.

## Discussion

### How is Hypersexuality Presented by Redditors with Bipolar?

Qualitative analysis of the 14 thematic categories inferred from keywords demonstrate that Redditors in the HiB-RC talk about hypersexuality within the wider context of bipolar symptomatology, where many keywords reference the fluctuating mood states which characterise the condition, as well as the feelings and emotions which reflect these mood states. There is wide variation in the lexical items that are used to describe sexual behaviours themselves, and the corpus also includes many words and phrases which describe judgement and feelings of shame. From keyword analysis alone we gain a unique insight into the feelings that are expressed in posts that reference hypersexuality, including lexical items such *debilitating, crippling, unbearable, exhausting, chaotic*, and *restless*. The key terms demonstrate that a number of other risk-taking behaviours are described in association with hypersexuality which is consistent with previous research, including recreational drugs, binge drinking, impulsive spending, and self-harm.^6,14,72^ The key terms also point to previously reported impacts of wider risk-taking behaviours such as sexually transmitted infections and diseases,^20,73,74^ debt,^75,76^ and suicidal ideation and attempts.^6,77,78^

The results yield significant insight into lived experiences of hypersexuality and the corollaries of these experiences. Hypersexuality is often described in relation to behaviour that is unsafe or risky, with Redditors describing that they can feel out of control of their hypersexuality and describing it as “something they wouldn’t normally do” – a finding also reported in Harvey et al.^6^ Analysis of the concordance lines related to sexual behaviours reveals a number of different behaviours that people engage with during periods of hypersexuality, including meeting with sex workers in high-risk situations, watching porn compulsively, using dating apps to engage in casual sex, infidelity in monogamous relationships, and having unprotected sex. Some Redditors describe self-management strategies that they use to mitigate the potentially harmful impacts of hypersexuality, such as using sex toys or a trusted partner to remove the risk of STIs, or deleting dating apps during a period of hypersexuality. The category of trauma and abuse includes terms such as *rape*, *sexual trauma* and *childhood sexual abuse (CSA)* and reveals multiple facets related to sexual trauma. Redditors describe being more hypersexual as an adult as a result of childhood trauma^22,79,80^ as well as experiencing sexual assault as an adult as a result of being in a more vulnerable situation due to hypersexuality.^18-20^ Further exploration of CSA as a risk factor for hypersexuality in bipolar would be beneficial in designing targeted interventions and to direct relevant services towards potentially at-risk populations.

### How Could the Findings of This Research be Used to Improve Clinical Practice for People with Bipolar?

The results suggest that discussion around hypersexuality needs to be normalised, both in a clinical context as well as more widely in society. Importantly, this includes the consistent provision of psychological therapy to people living with bipolar to provide a safe space where they can discuss their symptoms. There is a chasm between best practice for psychological therapies as described in the NICE guidelines^81^ and service user experiences. This is reflected by data presented in the guidelines, where audits conducted by South London and Maudsley NHS Foundation Trust and Manchester Mental Health and Social Care Trust show that rates of access to psychological interventions for eligible individuals with severe mental illness range between only 7% and 10%.^81^

The findings in this study related to shame and judgement are particularly salient as the results indicate that these feelings contribute to people being less inclined to discuss their experiences with healthcare professionals, with some Redditors reporting that they have only shared their sexual experiences on Reddit. The taboo around hypersexuality is also referenced in the category of therapy and treatment, where some Redditors describe healthcare professionals as being reticent to discuss hypersexuality or state that they feel too much shame to broach the topic themselves. This issue has also been reported in the wider literature.^82-85^ In their synthesis of qualitative interviews with healthcare professionals, Dyer and das Nair^86^ report that many participants do not feel they have the time, resources, knowledge or ability required to address complex issues such as sexuality. Previous research on the role of shame and guilt in hypersexual behaviour and sexual addiction (not specific to bipolar)^21,87^ indicates that “shame needs to be addressed when treating patients who struggle with hypersexual behaviour.”^21^ These studies suggest that a cycle of shame may develop, where hypersexual behaviours serve as a coping mechanism or a self-soothing behaviour to manage deep-seated or persistent feelings of shame. Ultimately the stigma surrounding hypersexuality means that people with this symptom may not be receiving the support they need, which could potentially contribute to unresolved trauma and delayed recovery.^19^ There have been previous calls to update the language used to refer to hypersexuality in bipolar in the DSM-5 to de-trivialise the symptom,^18,19^ and healthcare practitioners may benefit from improved training programmes – both to learn more about the symptom and to understand how to talk about hypersexuality in a non-stigmatising way. Consulting with lived-experience experts in developing these training programmes could foster a reciprocal learning process and ensure the training is both relevant and valuable from the perspective of service users. Read^88^ offers similar recommendations for enhancing the way clinicians address child abuse, emphasising the importance of mandatory training for mental health professionals. He states that this training should focus on both the skills needed to ask about abuse and the knowledge required to respond effectively. He advocates for teaching clinicians to ask objective, behaviourally-based questions about specific incidents, rather than broad questions like “Were you abused?.” Additionally, he suggests that training should cover managing a range of client responses, providing appropriate support, and ensuring safety. Read^88^ also highlights the need for training programmes to be tailored to local contexts and resources and stresses the value of involving mental health service users in developing policies, best practice guidelines, and training.

### Research Strengths

This research has employed corpus methods to provide valuable insights into the experiences of hypersexuality in individuals living with a bipolar diagnosis. By conducting large-scale qualitative analysis, we have made a significant contribution to this emerging field of study. Our approach efficiently identified key thematic categories within the broader topic of hypersexuality, offering insights into how these findings can inform clinical practice. Ultimately, our aim is for these findings to serve as recommendations to enhance access to support for individuals experiencing hypersexuality.

### Limitations

As with any analysis using social media data, we must assume the posts in our corpus are truthful. As noted by others,^89^ the stigma surrounding mental illness makes it unlikely that Reddit users would falsely report symptoms of a condition they do not have. However, response bias is a general issue in health research, and this limitation is not unique to studies using social media data. Any study that relies on self-reported information, whether through surveys, interviews, or online posts, must consider the potential for inaccuracies, as participants may consciously or unconsciously misrepresent their experiences. We must also recognise that the corpus may represent bias user demographics as Reddit users tend to be younger, more tech-savvy, based in the US and predominantly male.^90^ However, recent research which investigates the demographics of a corpus of Redditors who self-report bipolar suggests that more than half of the Redditors in their corpus are female with a less skewed distribution of age groups compared to average US Reddit users.^43,90^

We also note that due to the restricted set of hypersexuality keywords which was used to build the HiB-RC, the data presented is not fully representative of all experiences and perspectives on hypersexuality in individuals with bipolar across Reddit. It is likely that many Redditors discussed their experiences using vocabulary not captured by our pattern-matching expressions, meaning their posts would not have been retrieved.^41^

We acknowledge that reflexivity is an essential component of the research process. The authors’ perspectives, informed by both clinical and academic backgrounds, inevitably shape the interpretative lens through which data is analysed. The qualitative inductive approach used in our thematic coding process was an integral part of the reflexive research process. While this method allowed for a rich and nuanced exploration of lived experiences, it required ongoing critical awareness of the researcher’s interpretative role. Given the inherently interpretative nature of qualitative coding, alternative readings of the data are always possible. Furthermore, the reliance on publicly available Reddit posts, rather than direct engagement with participants, required careful consideration of context and meaning within the data.

This study has yielded various avenues for further research, including more in-depth investigation on the role of shame and judgement and further investigation into experiences of trauma and abuse as well as CSA in relation to hypersexuality. A linked important area of research could use a subreddit such as r/bipolarSOs (used by people in a relationship with someone who has bipolar) to understand how hypersexuality is experienced from an external perspective, which could yield insight into early detection and presentation of the symptom of hypersexuality.

## Conclusion

This study has provided valuable insights into how hypersexuality is discussed by individuals who report a diagnosis of bipolar on Reddit, shedding light on the lived experiences of this symptom. By employing corpus-assisted discourse analysis, we were able to identify key thematic categories and linguistic patterns related to hypersexuality. The findings reveal that hypersexuality is often described in the context of fluctuating mood states and sometimes framed by feelings of shame and judgement, which contribute to the stigma surrounding this issue. A number of Redditors expressed a sense of being out of control, with hypersexuality leading to harmful consequences such as sexually transmitted infections, encountering vulnerable situations, and relationship breakdowns.

Moreover, the study highlights the importance of normalising discussions around hypersexuality in both clinical and social contexts. The research signposts to the need for better training for healthcare professionals to address this symptom without perpetuating stigma. Current therapeutic practices may not be sufficient in addressing the complex emotional and behavioural issues associated with hypersexuality in bipolar, which may contribute to unresolved trauma and delayed recovery.

Our findings suggest that improving access to psychological therapies, particularly those that provide a safe space for open discussions about sexuality, is essential. The development of training programmes for clinicians, informed by lived experiences, could also foster a more compassionate and effective approach to supporting individuals with bipolar who experience hypersexuality. Additionally, the research has demonstrated the utility of corpus linguistic methods in exploring sensitive topics and shows that Reddit serves as a unique space where individuals are able to make disclosures they may not feel comfortable sharing elsewhere.
